# The Effect of UV Illumination on the Room Temperature Detection of Vaporized Ammonium Nitrate by a ZnO Coated Nanospring-Based Sensor

**DOI:** 10.3390/ma12020302

**Published:** 2019-01-18

**Authors:** Lyndon D. Bastatas, Phadindra Wagle, Elena Echeverria, Aaron J. Austin, David N. McIlroy

**Affiliations:** Department of Physics, Oklahoma State University, Stillwater, OK 74078, USA; lyndon.bastatas@okstate.edu (L.D.B.); pwagle@okstate.edu (P.W.); elena.echeverria@okstate.edu (E.E.); aaron.j.austin@okstate.edu (A.J.A.)

**Keywords:** nanospring, ammonium nitrate, gas sensor, room-temperature operation, UV-illumination

## Abstract

The effect of UV illumination on the room temperature electrical detection of ammonium nitrate vapor was examined. The sensor consists of a self-assembled ensemble of silica nanosprings coated with zinc oxide. UV illumination mitigates the baseline drift of the resistance relative to operation under dark conditions. It also lowers the baseline resistance of the sensor by 25% compared to dark conditions. At high ammonium nitrate concentrations (120 ppm), the recovery time after exposure is virtually identical with or without UV illumination. At low ammonium nitrate concentrations (20 ppm), UV illumination assists with refreshing of the sensor by stimulating analyte desorption, thereby enabling the sensor to return to its baseline resistance. Under dark conditions and low ammonium nitrate concentrations, residual analyte builds up with each exposure, which inhibits the sensor from returning to its original baseline resistance and subsequently impedes sensing due to permanent occupation of absorption sites.

## 1. Introduction

Detection and sensing of chemical hazards, improvised explosive devices, etc., is the first line of defense and the most effective approach for minimizing their threat to human health and safety. Toxic or dangerous agents produce vapors that, if accurately detected, serve as signatures of the products. For these reasons, it is imperative to develop sensors that can effectively detect harmful gases in real time at parts per billion concentrations, have few false positives, have fast recovery and are refreshable. Among different sensing platforms, semiconducting metal oxide-based sensors have garnered significant attention due to their electrical responsiveness to gas vapors, can be tailored to be selective to a target vapor analyte, good portability, low power consumption and low fabrication cost [1,2,3,4,5]. Zinc oxide (ZnO) is one of the most widely used metal oxide materials for chemical vapor sensors because of the combination of its optical and electrical properties, abundance in nature, non-toxicity, and bio-compatibility [4,6,7,8,9]. The response of a ZnO-based vapor sensor to a target analyte is a change in its conductance or resistance. Such changes in the resistance are attributed to analyte-induced variations of the width of the space charge near the surface of the semiconductor [9]. The width of the depletion layer is determined by the concentration of charge species adsorbed, as well as by the oxygen vacancies at the surface [10]. Furthermore, depending on the type of gas that is pulsed on the sensor, the band bending can either decrease or increase [11]. For n-type ZnO, a decrease in the band bending lowers the height of the intergrain barrier, decreases of the width of the depletion layer, and increases the probability of diffusion of carriers across grain boundaries, thus increasing the conductivity of the sensor [12].

To improve the sensitivity of ZnO-based sensors, they have been constructed as hybrid sensors [13,14,15], heterojunctions [16], field effect type sensors [17], and various nanostructures that have a high surface-to-volume ratio. Some of the architectural designs of nanostructures include nanowires [18,19], nanobelts [20], nanoflowers [21,22] and nanosprings [23,24,25]. Nanosprings have an accessible surface area of 200 m^2^/g, which is larger than that of the reported surface area of nanorods (~108 m^2^/g) [26], nanoflowers (~13 m^2^/g) [21] and nanobelts (~47 m^3^/g) [27]. Furthermore, an additional feature of these nanostructures is the presence of sites where two or more wires are in contact—electrical junctions. These junctions are critical to the conductivity of these systems [28]. A potential barrier develops at the junctions, which regulate the electron transport [29,30]. This implies that having greater junction density enhances the sensitivity of the sensor. Among the aforementioned nanostructures, nanospring assemblies contain relatively larger numbers of junctions due to their randomly oriented helical configuration.

Typically, ZnO and other types of metal oxide-based chemical sensors are operated at elevated temperatures (>300 °C) in order to increase hopping conduction and to enhance their surface reactivity through a redox reaction with the target analyte [23,31,32]. However, redox-based detection has several drawbacks. First, the redox process is not sufficiently selective; ergo, false positives can be problematic. Secondly, high-temperature operation is incompatible with surface functionalization for targeted detection. Finally, there is an energy cost with high-temperature operation. The alternative is to abandon redox-based detection in favor of surface functionalization-based detection at room temperature or slightly higher temperature. Unfortunately, metal oxides have poor conductivity within this temperature range due to low carrier hopping rates, which adversely affects their electrical responsiveness. UV irradiation of metal oxides is a viable replacement for thermal activation of carriers, and it enhances surface activity and analyte desorption of metal oxide sensors [12]. For example, Comini et al. [33,34] demonstrated enhanced CO and NO_2_ detection using SnO_2_-based sensor with 365 nm UV illumination. UV-assisted gas sensing was also conducted by Law et al. [35], who investigated the photochemical sensing of NO_2_ with SnO_2_ nanoribbon sensors at room temperature, Yang et al. [36] observed an enhancement in detecting CO using nano-TiO_2_ sensor, and Fan et al. [37] observed improved H_2_ sensing using polycrystalline ZnO with illumination, to name a couple.

The work presented herein is focused on detecting ammonium nitrate (AN) using a ZnO-based sensor, where AN is more challenging to work with compared to other gases, such as CO, NO_2_, etc., because of its low decomposition, even at 140–200 °C [38], thus making it incompatible with redox-based detection. There are only a few reports of electrical detection of vaporized ammonium nitrate, as tabulated in Table 1, and none so far have investigated the UV photo-enhanced detection of said analyte. This work aims to examine the effect of UV illumination on the detection of vaporized ammonium nitrate in ambient atmospheres at room temperature with the aid of lock-in amplification using ZnO-coated nanospring mats. 

## 2. Experimental Section

**Figure 1:**
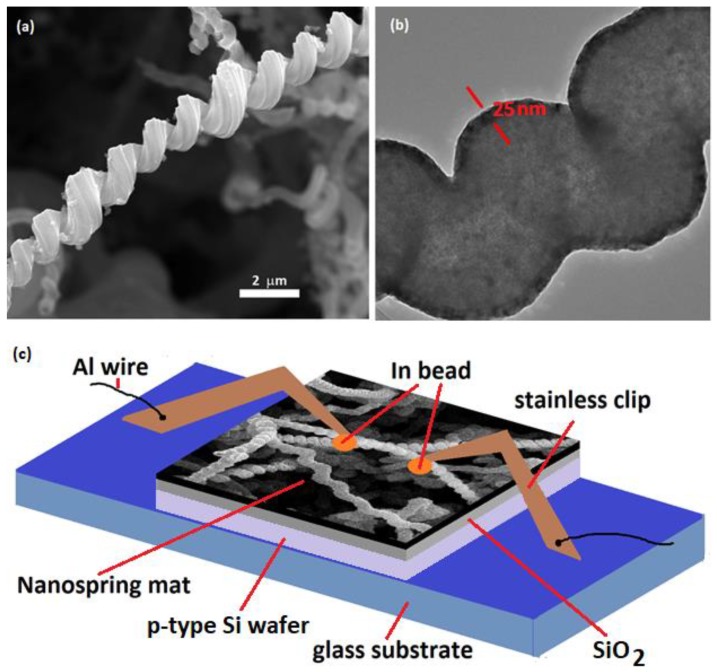
(**a**) Scanning electron microscopy (SEM) image of a bare silica-based nanospring grown for an hour and (**b**) transmission electron microscopy (TEM) image of nanospring coated with 25 nm ZnO; (**c**) schematic of the nanospring sensor mounted on a glass substrate, clipped with malleable indium beads to provide good contact.

The fabrication of silica-based nanospring (NS) ensembles has been reported in detail by McIlroy et al. [42] and Wang et al. [43], where the randomly oriented silica nanosprings were fabricated by a catalytic vapor-liquid-solid (VLS) process. In the present study, the nanosprings were grown on a 1 cm^2^ silicon wafer with thermal oxide layer, which in turn was coated with a thin layer of gold (~15 nm) that served as the catalyst for VLS growth of the nanosprings. Nanospring growth was performed in a custom-built reactor at 370 °C at atmospheric pressure with a constant flow of oxygen, nitrogen and a proprietary Si precursor for approximately an hour. This produces a ~60-nm-thick mat of silica nanosprings. A picture of bare silica nanospring mats is shown in Figure 1a.

The nanospring mats were then coated with a conformal layer of ZnO by atomic layer deposition (ALD) in a system from Okyay Technologies. The ALD process consists of 100 cycles, where each cycle consisted of a dose of diethylzinc, pump down and N_2_ purge, a dose of DI water, and a pump down and N_2_ purge. The ZnO film thickness of the calibration blank that was run simultaneously with the NS sample was determined by ellipsometry (J. Woollam, Lincoln, NE, USA), which yielded a film thickness of approximately 25 nm. The thickness of the ZnO coating on the NS was determined by transmission electron microscopy (TEM, JEM-2100, JEOL USA, Inc., Peabody, MA, USA), Figure 1b.

**Figure 2:**
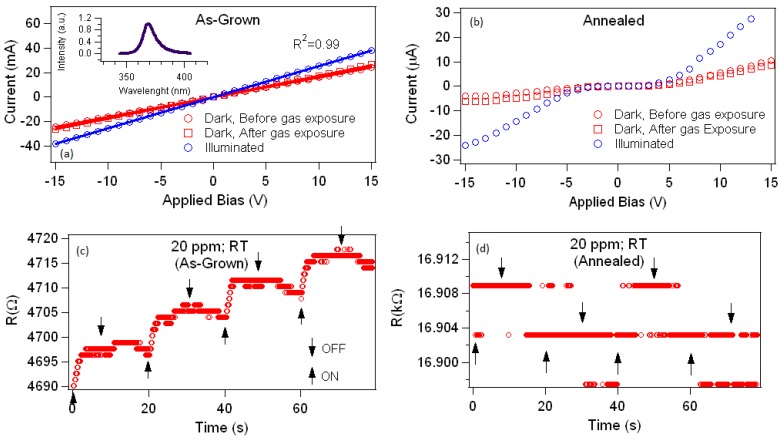
I–V curves under dark and UV-illuminated conditions of (**a**) as-grown and (**b**) thermally annealed ZnO-coated nanospring sensors in air. Inset of Figure 2a is the spectrum of the UV light that was used to illuminate the sensor. The solid line indicates the linear fitting. The linear feature of the I–V-curve in the as-grown ZnO-coated nanospring sensor indicates an Ohmic contact, while the non-linear nature of the curve after thermal annealing indicates a Schottky-like contact. The nature of contacts is preserved after gas exposure. Comparison between the response of the (**c**) as-grown and (**d**) annealed nanospring sensor when exposed to 20 parts per million of the target gas analyte.

The DC resistance of the sensor was determined from I–V curves acquired using a Keithley 2400 source/meter (Cleveland, OH, USA) under dark conditions and with UV illumination, where the sample bias was swept between ±10 V. A StellarNet LED (Tampa, FL, USA) with a peak intensity at 369 nm and ~17 mW of power (see inset of Figure 2a) was the UV light source used to illuminate the sensor. Two indium beads were used as contact pads with the sensor to obtain Ohmic contact between the ZnO coated NS and the electrodes. A schematic of the sensor is presented in Figure 1c. In a way, the architecture resembles that of a field effect transistor. Indium beads were used as the metal contacts because they are soft, malleable and create good electrical contacts with the highly non-uniform surface topology of the nanospring mat. Using the stainless clips to hold the beads in a place-and-clip manner ensures good electrical contacts. We found these contacts to be the most reliable for use in ensembles of 1-D nanostructures, as well as being inexpensive. The resistance of the electrodes is less than 1 Ω; hence, they are negligible compared to the sensor resistance.

The details of the gas sensing test station are described in detail in Reference [41]. Measurements were performed by exposing the fabricated sensor with vaporized AN at room temperature in ambient conditions, either under dark conditions or with UV-light illumination. The sensor was connected in series with a resistor to form a voltage divider. A lock-in amplifier (Stanford Research 510, Sunnyvale, CA, USA) was used to monitor the change in the voltage across the sensor. A function generator was used to modulate the sample bias and as a reference signal for the lock-in amplifier. The AN powder was sealed in glass flask on a hot plate and heated to produce a range of vapor pressures having temperature of 80–100 °C. The dosing of AN vapor was regulated by a Teflon solenoid valve teed into a constant 30 m/s stream of filtered dry air (carrier gas). The airflow produced a weak Venturi effect that assists with the extraction of AN from the flask. 

## 3. Results

### 3.1. Nanospring SEM and TEM Image

The morphology of the silica nanosprings and the ZnO coating were determined with scanning electron microscopy (SEM, FEI Quanta 600 FEG, Thermo Fisher Scientifics, Hillsboro, OR, USA) and transmission electron microscopy (TEM). Figure 1a is an SEM image of a bare silica nanospring grown. The size of the silica coil varies from tens to hundreds of nanometers and with lengths from hundreds of microns to submillimeters. The network of ZnO-coated nanospring has an accessible surface area of 200 m^2^/g. Figure 1b is a TEM image showing the thickness of the ZnO film coating, which is approximately 25 nm. The grain sizes for the as-grown ZnO coating are ≤15 nm, as reported elsewhere [41].

### 3.2. Electrical Properties of Nanospring Mats with UV Illumination and the Sensing Response of As-Grown and Annealed Samples

Presented in Figure 2a,b are the current-voltage (I–V) curves of the as-grown and annealed sensors, respectively, under dark and UV-illuminated conditions. A linear behavior is observed in the as-grown NS sensor, which is indicative ofan Ohmic contact between the ZnO-coated NS mat and the electrodes. Under dark conditions, the resistance of the as-grown sensor is 585 Ω, and this drops to 370 Ω with UV illumination. The illumination-induced drop in resistance is due to an increase in carrier concentration, where either carriers are freed from intrinsic traps, electron-hole pairs are created, or photoinduced desorption of oxygen from the surface [37]. After the gas sensing experiment, the electrical properties of the sensor were again tested, and no significant difference of the I–V curve was observed (Figure 2a), thus the Ohmic nature of the contacts is preserved. The resistance of the sensor, however, increases slightly to 600 Ω, which we attribute to incomplete desorption of AN.

Figure 2b shows that the I–V curve of the sensor thermally annealed to 500 °C in air became non-linear and accompanied by a drop (rise) of three orders of magnitude in current (resistance) relative to the as-grown sensor for the same applied bias. The deviation from the linearity of the I–V curve reflects the Schottky nature of the contact. The increase in the resistance is due to a decrease of charge carrier concentration, increase of ZnO grain sizes, and formation of grain boundaries that trap mobile carriers [44,45,46]. 

The impact of defect states that are abundant in the as-grown ZnO-coated nanospring favorably forms Ohmic contacts between the semiconductor and indium (Figure 2a) or even with gold electrodes. The Ohmic contact minimizes the resistance and provides a large number of conductive paths for the effective and rapid transport of charge carriers. Although some studies have demonstrated that Schottky contacts can enhance the sensitivity of the sensors, the operation requires either application of strain to create a piezopotential [47,48], or reverse biasing [49], which were not employed in this study. In our case, we applied AC signal to drive the sensor with lock-in detection.

The as-grown and annealed ZnO-coated nanospring mats were used as the sensing material for detecting AN vapor. Figure 2c,d shows the responses of the as-grown and annealed sensors, respectively, when exposed to 20 parts per million (ppm) of ammonium nitrate vapor. As shown, the as-grown sample with Ohmic contacts is more responsive compared to the annealed sensor, which has Schottky-like contacts between the nanosprings and the In bead. However, the lower sensitivity of the annealed sensor cannot be solely attributed to the contacts.

### 3.3. Effect of UV Light on the Sensing of Ammonium Nitrate in Air

**Figure 3:**
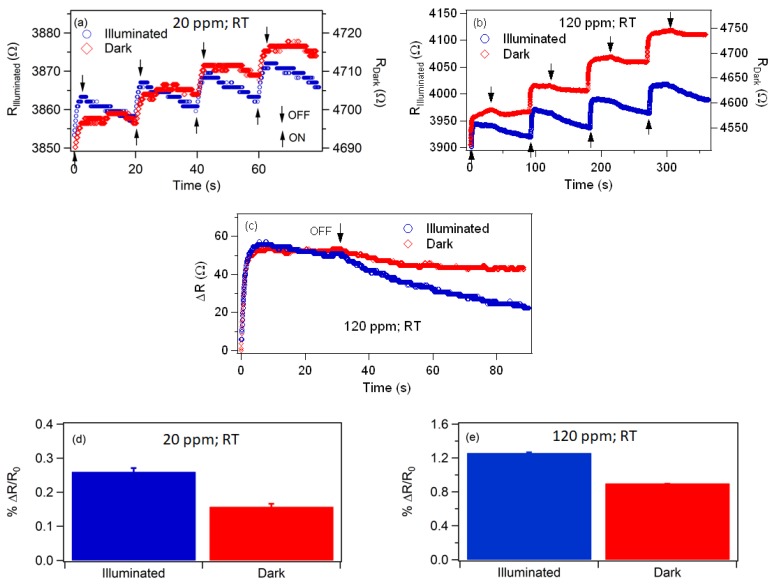
The stability response showing the variation of the resistance of the (as-grown) sensor in dark (red markings) and illuminated (blue markings) conditions when exposed to (**a**) 20 ppm and (**b**) 120 ppm of AN vapor using In as contact electrodes. The upward arrows indicate the moment when the vapor was introduced and the downward arrows when the gas was cut off. (**c**) A single pulse of AN dosing showing the change of the resistance and recovery as a function of time when exposed with 120 ppm of AN vapor. The sensitivity of the sensor when exposed to (**d**) 20 ppm and (**e**) 120 ppm of AN vapor. The change in resistance was determined using Equation (1) by taking R_A_ as the resistance immediately before the pulse and R_G_ as the resistance after the pulse.

Figure 3a,b shows a comparison of the as-grown sensor’s response with exposures to 20 ppm and 120 ppm of vaporized AN, respectively, with and without UV illumination. It can be observed that the baseline resistance changes with gas exposure, and the drifting of resistance is more prominent under dark conditions than with UV illumination. Also note that, prior to exposure to AN, the sensor was allowed to stabilize. This suggests that the drifting of the resistance is due to incomplete desorption of AN from the surface of the sensor. As can be seen in Figure 3c, although the change in the resistance under dark conditions and with UV illumination are approximately equal, the recovery of the sensor is faster with UV illumination. Fitting the recovery side of the data of the UV illuminated sensor with an exponential function, we extracted recovery time constants of 30 s and 90 s for 20 ppm and 120 ppm, respectively. A similar fit of the recovery of the sensor under dark conditions and when exposed to 120 ppm gives a time constant of 102 s, but this could not be performed on the data for 20 ppm of AN due to the plateauing of the signal, i.e., the signal ceases to return to the baseline resistance in the timespan between doses, as seen in Figure 2c. This plateauing under dark conditions and low concentrations supports the conclusions that a fraction of the adsorbed AN does not completely desorb, and that UV illumination stimulates desorption. A similar response was observed when the indium beads were replaced with gold pads. Please note that even with lock-in amplification, a change in the resistance of the sensor was detected during the exposure of the gas analyte, which affirms the challenge in detecting AN electrically.

By convention, the sensitivity of the sensor can be determined as

(1) $$\%\frac{\Delta R}{R_{o}}=\frac{\left|R_{G}-R_{A}\right|}{R_{A}}\times 100\%$$

where *R_G_* is the maximum resistance of the sensor upon gas exposure and *R_A_* is the resistance prior to pulsing of vapor. Using Equation (1), it was observed that the sensitivity is enhanced with UV-illumination, as shown in Figure 3d,e. That is, at 20 ppm, the percent increase of resistance is 0.15% in the dark, but increases to 0.26% when illuminated. At higher AN vapor concentration (120 ppm), the values increase to 0.9% and 1.25% under dark and illuminated conditions, respectively. Based on Equation (1), both cases correspond to at least a 20% enhancement of the response with illumination. 

## 4. Discussion

**Figure 4:**
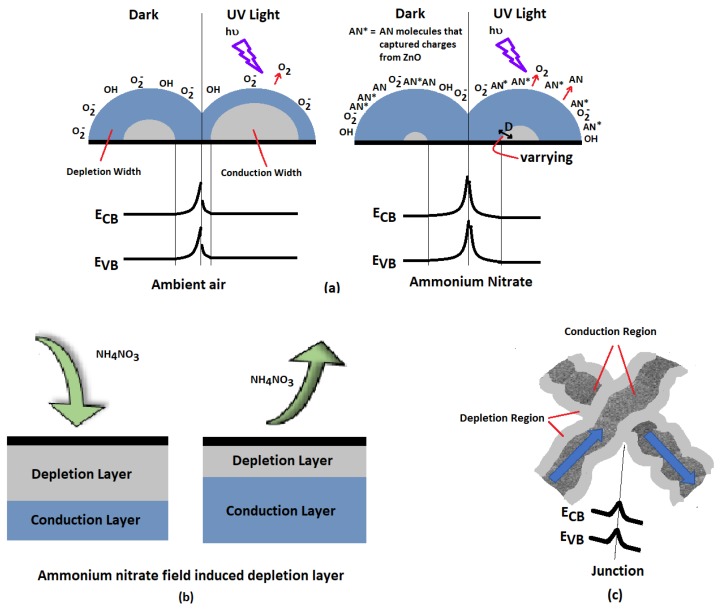
Schematics of the ammonium nitrate detection mechanism. (**a**) Left: Absorption/desorption of ambient air. Right: Adsorption/desorption of ammonium nitrate. The oxidizing component of ammonium nitrate captures electrons, thereby increasing the width of the depletion layer. The width, D, of the depletion layer with AN exposure can either decrease, increase or stay the same with UV illumination in comparison to dark conditions. The critical width of the depletion layer for achieving optimal response depends on the materials and operational parameters. (**b**) The dipole field of AN causes a redistribution of charges near the ZnO surface, and thus affect the width of depletion layer. (**c**) The junction of nanospring networks develops a potential barrier with AN vapor exposure. The blue arrows show the direction of the electronic conduction.

The detection of AN vapor using ZnO-coated (n-type) nanospring sensors arises from the localized effect of charge transfer and the at-a-distance field effect of the AN, as illustrated in Figure 4 [41,50,51]. The interaction between the oxygen vacancies and the adsorbed AN nitrate changes the width of the depletion layer near the surface due to charge transfer between the two. In addition, the dipole field of the AN molecule [52] causes redistribution and polarization of charges on the surface [53]. Furthermore, the potential barrier at the junctions between nanosprings within the network is modified by AN adsorption [29]. These mechanisms obstruct and narrow the conducting path for electron transport; hence, the resistance of ZnO-coated nanosprings increases with exposure to AN vapor.

**Figure 5:**
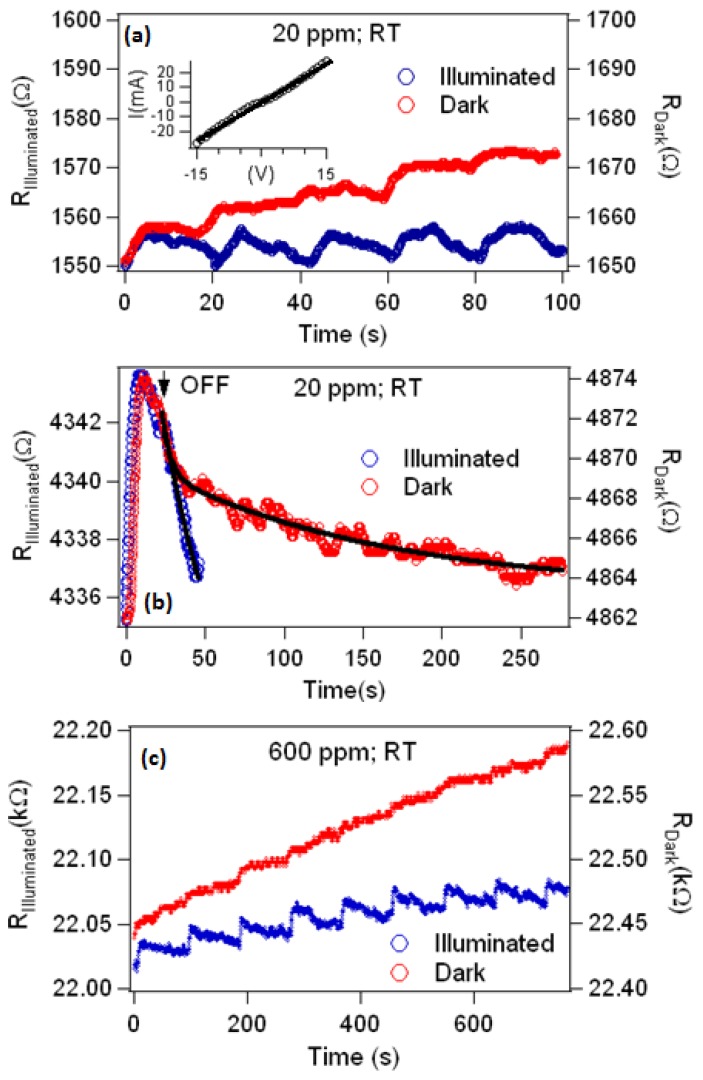
(**a**) Electrical response of (as-grown) ZnO-coated nanospring mats using gold pads as the contact electrodes when exposed with 20 ppm of vaporized ammonium nitrate. The inset shows an almost linear I–V relationship. (**b**) Comparison of the resistance profile of the (as-grown) sensor with a single pulse/exposure to 20 ppm AN vapor in dark and illuminated conditions where the sensor was given sufficient time to recover. (**c**) Representative data (annealed sensor, 600 ppm of AN vapor) showing that the change in resistance of the sensor is larger when UV-illuminated compared to the dark condition.

Vapor adsorption can either be chemisorption (strong) or physisorption (weak). The former involves formation of chemical bonds between gas molecules and metal oxide surface atoms, whereas the latter is due to Van der Waals interaction between gas molecules and the metal oxide. One way to distinguish one from another is through the desorption time scale. The former process requires a relatively longer period of time, while the latter is a faster process [54]. Figure 5b shows a longer time scale response for the as-grown sample when exposed with 20 ppm of AN vapor. As can be seen, the decay of the resistance can best be fitted with a double exponential function that captures the fast and slow decay components of the relaxation. The fast process is described with a time constant, τ_f_, and the slow process with a time constant, τ_s_. In the case of the dark condition, the time constants extracted from the resistance profile of the sensor by performing the fitting are τ_f_ = 0.4 min and τ_s_ = 15.4 min, while for that of the UV-illuminated sensor yields τ_f_ = 0.04 min and τ_s_ = 0.9 min. Clearly, the recovery of the sensor is accelerated with UV illumination. The fast and slow processes in Figure 5b are due to different mechanisms. Presumably, the quick decay of the resistance indicates that the AN vapor physically adsorbed onto the surface of ZnO, while a longer time is an indication of chemical adsorption. With UV illumination, the surface activity is enhanced, and the activation energy for desorption reaction is lowered [55]. The electrons that absorb sufficient photon energies are excited from the valence band and donor states, and thus the concentration of electrons in the conduction band increases. The photogenerated electrons can be captured by the oxidizing components of the AN molecules. The photogenerated holes in the valence band can move towards the surface and facilitate the neutralization of the ionized molecules, leading to the desorption of the molecules from the ZnO surface. In addition, since the activation energy of the desorption reaction decreases, the desorption of the molecules accelerates [55]. 

The effects of illumination are two-fold. First, it stimulates desorption of AN, thereby accelerating the recovery rate of the sensor. Secondly, it minimizes the drifting of the baseline resistance of the sensor, which has been observed previously [37,56,57]. The linear increase of the baseline that is observed in Figure 3a,b and Figure 5a supports our earlier conclusion that residual AN remains on the ZnO surface after each exposure. This increases the possibility of using the sensor to quantify chemical interactions with the surface or probe surface defects of ZnO. The fact that UV illumination increases the conductivity of the ZnO-coated NS and decreases the concentration of residual AN that remains after each exposure suggests about the bonding of AN to ZnO defects, where a density functional theory simulation of gas absorption on ZnO nanotubes found that the gas adsorption energy is larger for surfaces with oxygen vacancies than for those without [50]. The higher concentration of carriers (electrons and holes) and their higher mobility neutralize shallow traps that are accessible to valence states of AN and serve as weak bonding sites. The residual AN is therefore bound to energetically deeper defects, and is possibly chemisorbed, rather than being physisorbed. 

It is worth mentioning that in some instances, the change in the resistance with UV illumination was 10% larger than under dark conditions (e.g., shown in Figure 5c), but without consistency. Nevertheless, this suggests that UV illumination could increase the sensor’s sensitivity, as was observed for some gases [55,58,59]. For instance, by using different UV light intensities, Chinh et al. [55] reported that optimal response of a sensor constructed with indium oxide nanostructures to nitric oxide vapor occurred at an intermediate intensity and attributed it to a critical depth of the surface depletion layer. They proposed that critical depletion depth depends on the concentration of charge carriers, temperature and concentration of the gas analytes.

Similarly, we hypothesize that the critical depth of the depletion layer stems from the dynamics of the ionized bulk donor concentration, *N*, gas analyte concentration, *A_c_*, and surface potential due band bending, φ. Provided that the thickness of the ZnO layer is larger than the width of the space charge region, the depletion depth, *D*, is given by [60]:

(2) $$D=\sqrt{\frac{2\epsilon \varphi }{qN}}$$

where ϵ is the dielectric constant of the semiconductor and *q* is the electronic charge. The resistance of the sensor increases with the increasing depth of the depletion region. Therefore, to optimize the response of the sensor, the effect of UV illumination, in conjunction with analyte exposure at a specific concentration, must be such that either the band bending potential increases, the concentrations of carriers and ionized donors at the bulk decrease, or a combination of both. The surface band bending is affected by the near-surface charge carrier concentration and the field effect produced by the analyte. In cases where an increase of bulk donor concentration occurs [61] (e.g., concentration oxygen vacancies increases), this will be commensurate with the analyte-induced surface band bending, so the effect of UV illumination will be negated. Consequently, the maximum sensor response to the analyte will not be obtained. In addition, since light can also excite electrons to overcome barriers, the intensity and photon energy must also be carefully considered in order to harness the effect of the junctions. Highly energetic electrons can easily overcome barriers, to the point that the potential barriers at junctions no longer impede conduction, thereby rendering the sensor less sensitive with exposure to AN vapor.

In effect, we have a set of materials (carrier concentration, defect density, etc.) and operational parameters (wavelength of light source, intensity of the light source, sensor temperature, bias, etc.) that have to be tuned to work together to optimize the response of the sensor to the analyte—ammonium nitrate in this study. Finally, UV illumination is a tool for refreshing the sensor. It does not appear to be too effective at high analyte concentrations, but is very effective at low concentration, which also happens to be the operational target of this study.

## 5. Conclusions

We examined the effect of UV illumination on the responsiveness of a ZnO-coated nanospring sensor to ammonium nitrate vapor operated at room temperature. Utilizing lock-in detection, we were able to detect low ppm concentrations of ammonium nitrate and identify the mechanisms by which UV illumination enhances the response and refreshing of the ZnO NS sensor. Specifically, UV illumination reduces surface band bending, thereby promoting better intergrain charge transport, which decreases the drift of the sensor’s baseline resistance. UV illumination reduces the sensor recovery time between detection events by shortening the decay time of the signal. In addition, it stimulates desorption of the analyte, which improves the reproducibility of the sensor signal and the profile of the signal. UV illumination did not consistently increase the sensor response, but there is evidence to suggest that judiciously choosing the wavelength of the UV light could enhance the sensor signal. Consequently, obtaining the maximum sensor response to the analyte was not achieved. Future work will focus on enhancing the responsiveness of the ZnO NS sensor through materials design (resistance, carrier concentration, defect density, etc.) and optimization of the operational parameters (wavelength of light source, intensity of the light source, sensor temperature, bias, etc.) in concert. 

## Figures and Tables

**Table 1:** Studies on AN detection through electrical measurements.

Structure	Concentration	Temperature	Reference
Orthogonal sensor made of SnO_2_ and ZnO	Not Provided	150–400 °C	[39]
dPPV/zeolite composites	337 ppm	RT	[40]
ZnO-coated nanospring mat with lock-in amplification	20 ppm	RT	[41]
